# Metabolomic Profiling Reveals the Difference on Reproductive Performance between High and Low Lactational Weight Loss Sows

**DOI:** 10.3390/metabo9120295

**Published:** 2019-12-04

**Authors:** Liang Hu, Lianqiang Che, Chen Wu, Mihai Victor Curtasu, Fali Wu, Zhengfeng Fang, Yan Lin, Shengyu Xu, Bin Feng, Jian Li, Yong Zhuo, Peter Kappel Theil, De Wu

**Affiliations:** 1 Key Laboratory for Animal Disease-Resistance Nutrition of China Ministry of Education, Institute of Animal Nutrition, Sichuan Agricultural University, No. 211, Huimin Road, Wenjiang District, Chengdu 611130, Sichuan, China; huliangsau90@hotmail.com (L.H.); clianqiang@hotmail.com (L.C.); 18283581065@163.com (C.W.); 15008318848@163.com (F.W.); fangzhengfeng@hotmail.com (Z.F.); linyan936@163.com (Y.L.); shengyu_x@hotmail.com (S.X.); fengb123d@163.com (B.F.); lijian522@hotmail.com (J.L.); zhuoyong@sicau.edu.cn (Y.Z.); 2 Department of Animal Science, Faculty of Science and Technology, Aarhus University, DK-8830 Tjele, Denmark; mihai.curtasu@anis.au.dk (M.V.C.); peter.theil@anis.au.dk (P.K.T.)

**Keywords:** lactational weight loss, metabolites, oxidative stress, reproductive performance, sow

## Abstract

Sows suffering excess weight loss during lactation may delay weaning to estrus interval (WEI) and have a detrimental effect on subsequent reproductive performance, however, the underlying mechanism is not completely clear. Therefore, the goal of this study was to investigate physiological profiles manifested in plasma originating from high (HWL) and low lactational weight loss (LWL) sows. The plasma biochemical parameters, hormones, antioxidant parameters, and milk compositions were assessed. Furthermore, plasma metabolites were analyzed using ultrahigh-performance liquid chromatography/time-of-flight mass spectrometry in positive and negative ion modes. Results showed that HWL sows had a lower feed intake and higher lactational weight loss and prolonged WEI, but had similar litter performance and milk composition compared to LWL sows. These changes were associated with lower plasma insulin-like growth factor 1 and higher fibroblast growth factor 21 levels in the HWL sows. Moreover, HWL led to a severe oxidative stress and metabolic damage, as accompanied by excessive protein breakdown and lipids mobilization at weaning. Metabolomic analysis revealed differences in 46 compounds between HWL and LWL sows, and the identified compounds were enriched in metabolic pathways related to amino acids metabolism, fatty acids oxidation metabolism, bile acids biosynthesis, and nucleoside metabolism. These results provide the evidence for physiological mechanism in sows with excessive lactational weight loss that delayed the WEI. Metabolomic data provides essential information and gives rise to potential targets for the development of nutritional intervention strategies.

## 1. Introduction

Modern lactating sows have a large demand for energy and nutrients for supporting the large litter. However, the feed intake of lactating sows is often not sufficient to fulfil the energy demands for maintenance and milk production [1,2]. The discrepancy between a large nutrient demand and insufficient nutrient intake leads to mobilization of their body reserves, to keep sufficient milk production for suckling pigs during the lactation period, ultimately resulting in a high body weight loss (HWL) at weaning [3,4]. Body mobilization is positive for the offspring, but might be harmful to the sow [4]. Numerous studies have shown that excessive weight loss in sows negatively influences weaning to estrus interval (WEI), subsequent farrowing rates, total born litter sizes and the variation of birth weight [5,6,7], due to insufficient restoration of follicle development, affecting ovulation rate and embryo quality [8,9]. The change of metabolic status may be the main factor contributing to the restricted follicular development of HWL sows. Previous studies reported that sows with excessive weight loss are associated with low plasma glucose, insulin and insulin-like growth factor-1 (IGF-1) concentrations and high non-esterified fatty acids (NEFA) concentrations [7]. However, the causes and under lying mechanisms of compromised subsequent reproductive performance by excess lactational weight loss are not completely clear.

Plasma can be used as a metabolic fingerprint that provides a visual result of the metabolic events and reveal changes in metabolic pathways under various nutritional or physiological conditions [10]. Metabolomic, as an emerging analytical approach in systems biology, refers to the global analysis of all small molecular metabolites of an individual [11]. The global metabolic profile can directly reflect the final result of the effects of a variety of factors, such as physiological, genetic, and environmental factors [12]. Therefore, metabolomic is an ideal tool to explore the various effects of excessive weight losses on plasma metabolites that result from catabolic processes in lactating sows.

In addition, due to the physiological status of sows from anabolism to catabolism during late gestation and lactation, increased free radicals production leads to decreased antioxidant capacity and results in increased oxidative damage [13]. A previous study has shown that dairy cows with a higher loss of body condition score are more sensitive to oxidative stress [14]. In humans, a close relationship between high body weight loss and oxidative stress has also been demonstrated, most importantly, the incidence of some metabolic diseases might be induced by excessive oxidative stress [15,16]. However, there is no study available to determine the status of oxidative stress in sows suffering from excessive lactational weight loss.

In the current study, therefore, we aimed to elucidate differences in the metabolome of HWL sows versus low weight loss (LWL) sows using ultrahigh-performance liquid chromatography/time-of-flight mass spectrometry. Furthermore, the effects of body weight loss during lactation on biochemical parameters, hormones and antioxidant parameters of multiparous sows were also determined. The results of this study will be of great importance for understanding the underlying physiological difference between HWL and LWL sows, and this may be beneficial to generate new hypothesis and to alleviate the negative consequences of excess body mobilization in the future.

## 2. Results

### 2.1. Sow Performance and Body Condition

The HWL sows had lower average daily feed intake (ADFI) than the LWL sows (Table 1, *p* < 0.05). However, the reproductive parameters did not differ between HWL and LWL sows (*p* > 0.05). The body condition at parturition did not differ between HWL and LWL sows (Table 2, *p* > 0.05), whereas HWL sows had lower (*p* < 0.05) body weight, fat mass, and protein mass than that of LWL sows at weaning, resulting in higher body weight loss and back fat loss during lactation compared with LWL sows (*p* < 0.05). Compared with LWL sows, HWL sows had a higher lactation fat loss (9.17%, *p* < 0.001) and protein loss (14.24%, *p* < 0.001).

### 2.2. Plasma Biochemical Profiles, Colostrum and Milk Composition

**Figure 1:**
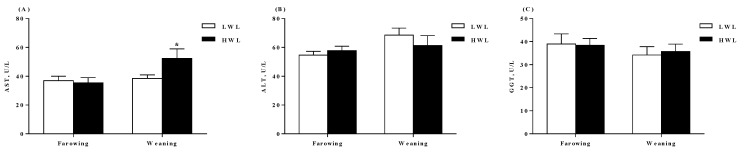
Plasma AST (**A**), ALT (**B**), and GGT (**C**) activities of sows with a low or high lactational weight loss at farrowing and weaning, respectively. Data are expressed as the mean ± SEM. Sows were regarded as the experimental units, *n* = 10 for each group. The differences between groups were indicated by asterisk (*p* < 0.05). LWL, low weight loss; HWL, high weight loss; ALT, alanine aminotransferase; AST, aspartate aminotransferase; GGT, gamma-glutamyl transpeptidase.

The colostrum and milk composition did not differ between HWL and LWL sows (Table 3, *p* > 0.05). Moreover, the plasma biochemical profiles did not differ (Table 4, *p* > 0.05) between HWL and LWL sows, except the NEFA concentration which was higher in HWL than in LWL sows at weaning (*p* < 0.05). In addition, HWL sows had higher AST activity than the LWL sows (Figure 1A, *p* = 0.022) at weaning. No evidence for difference (*p* > 0.05) in plasma ALT (Figure 1B) and GGT (Figure 1C) concentrations between the two groups were observed.

### 2.3. Plasma Antioxidant Parameters

**Figure 2:**
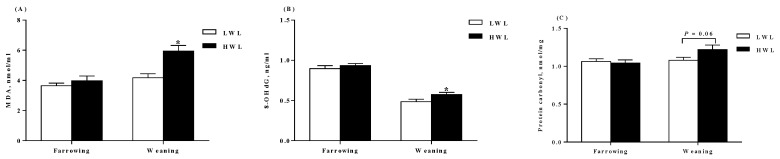

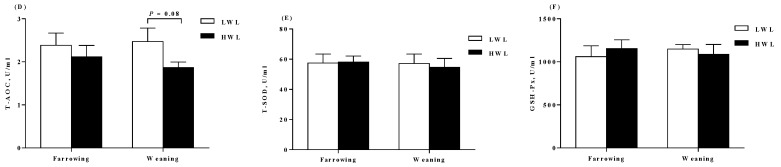
Plasma antioxidant indicators of sows with low or high lactational weight loss. MDA (**A**), 8-OHdG (**B**), protein carbonyl (**C**), T-AOC (**D**), T-SOD (**E**), and GSH-Px (**F**) in plasma at farrowing and weaning, respectively. Data are expressed as the mean ± SEM. Sows were regarded as the experimental units, *n* = 10 for each group. The differences between groups were indicated by asterisk (*p* < 0.05). LWL, low weight loss; HWL, high weight loss; T-SOD, total superoxide dismutase; MDA, malondialdehyde; GSH-Px, glutathione peroxidase; 8-OHdG, 8-hydroxy-deoxyguanosine; T-AOC, total antioxidant capacity.

Plasma levels of total superoxide dismutase (T-SOD), malondialdehyde (MDA), protein carbonyl, glutathione peroxidase (GSH-Px), total antioxidant capability (T-AOC), and 8-hydroxy-deoxyguanosine (8-OHdG) on days of farrowing and weaning are shown in Figure 2. The HWL sows had higher MDA (*p* < 0.05, Figure 2A) and 8-OHdG (Figure 2B) concentrations than the LWL sows at weaning. Moreover, the HWL sows tended to increase protein carbonyl concentration (*p* = 0.06, Figure 2C) at weaning. However, the HWL sows tended to decrease T-AOC (*p* = 0.08, Figure 2D) at weaning. There was no difference (*p* > 0.05) in plasma levels of T-SOD (Figure 2E) and GSH-Px (Figure 2F) neither at farrowing nor at weaning.

### 2.4. Plasma Hormone Determination

**Figure 3:**
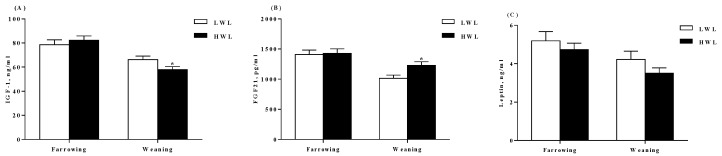
Plasma concentrations of fibroblast growth factor 21 (FGF21), insulin-like growth factor 1 (IGF1) and leptin in sows with low or high lactational weight loss. FGF21 (**A**), IGF1 (**B**), and leptin (**C**) in plasma at farrowing and weaning, respectively. Data are expressed as the mean ± SEM. Sows were regarded as the experimental units, *n* = 10 for each group. The differences between groups were indicated by asterisk (*p* < 0.05). LWL, low weight loss; HWL, high weight loss.

There were lower IGF-1 concentrations (*p* < 0.05) at weaning (Figure 3A), while higher fibroblast growth factor 21 (FGF21) concentrations (*p* < 0.05) in plasma of HWL sows as compared to that of the LWL sows (Figure 3B). However, plasma leptin concentrations did not differ (*p* > 0.05) between the two groups (Figure 3C).

### 2.5. Plasma Metabolic Profiling Based on Ultrahigh-Performance Liquid Chromatography/Time-of-Flight Mass Spectrometry

**Figure 4:**
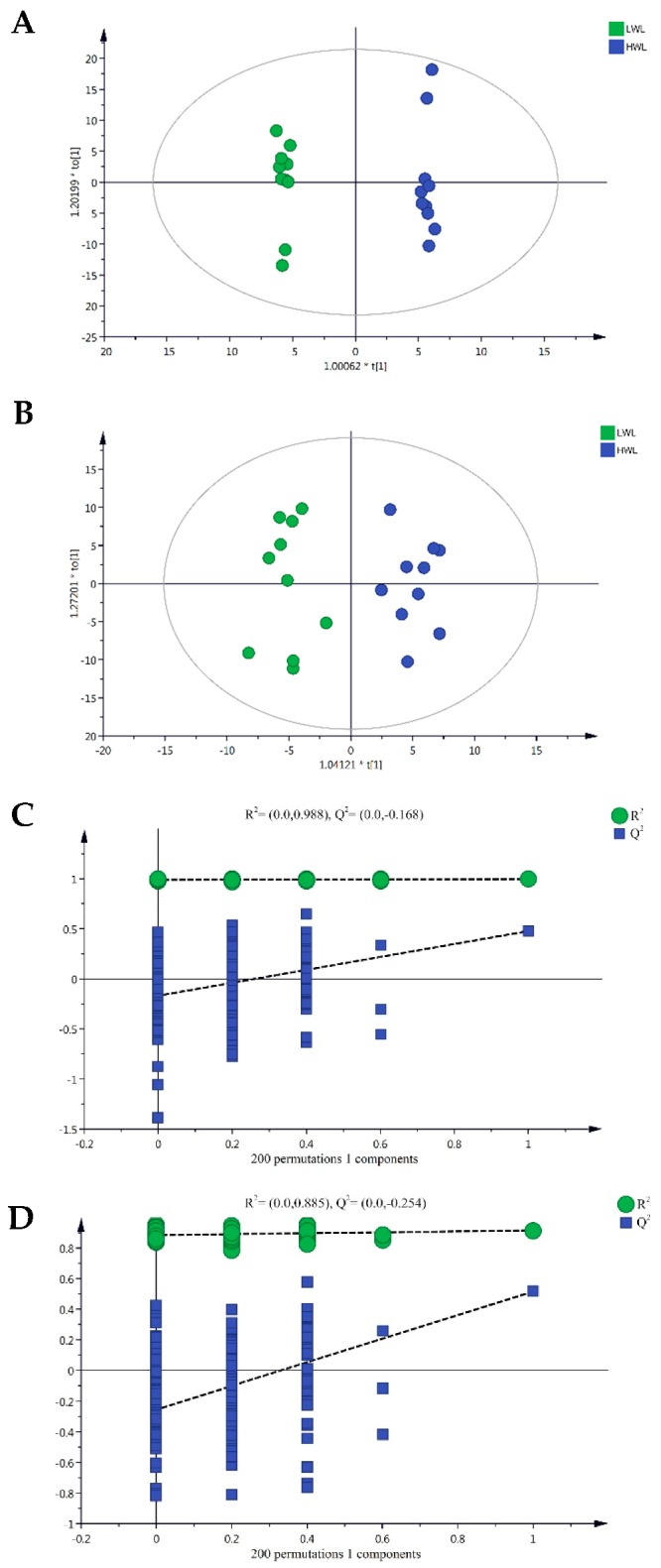
Orthogonal partial least squares discriminant analysis (OPLS-DA) score plots comparing HWL with LWL sows in positive electrospray ionization mode (ESI^+^) and negative electrospray ionization mode (ESI^−^) metabolomics profiles of plasma (Panels (**A**,**C**) are ESI^+^, Panels (**B**,**D**) are ESI^−^, respectively). LWL, low body weight loss; HWL, high body weight loss.

Plasma samples from the 10 HWL sows and 10 LWL sows were analyzed in both positive and negative ionization mode. Representative base peak chromatograms (BPC) were shown in Figure S1. Principal component analysis (PCA) and orthogonal partial least squares discriminant analysis (OPLS-DA) were employed to visualize the LC-MS dataset and display the similarities and differences among samples in the study. No significant difference between samples was observed in unsupervised PCA analysis when all features were used (Figure S2). To further investigate the discrimination between HWL sows and LWL sows, a supervised OPLS-DA analysis was then performed. The OPLS-DA score plots (Figure 4A,B) show separation between the HWL sows and LWL sows by the first principal component in both positive and negative modes. The OPLS-DA fit criteria were calculated as follows: R^2^X (cum) = 0.465, R^2^Y (cum) = 0.997, Q^2^ (cum) = 0.477 in positive mode and R^2^X (cum) = 0.250, R^2^Y (cum) = 0.915, Q^2^ (cum) = 0.516 in negative mode (Table S1). Both R^2^Y and Q^2^ values were greater than 0.4, indicating that the model was stable and reliable. The Q^2^ intercept values were less than 0.05, indicating that there was no overfitting (Figure 4C,D).

**Figure 5:**
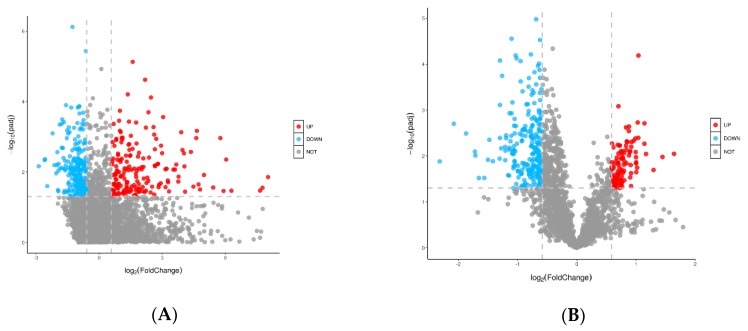
Volcano plots showing the distribution of all metabolites based on their fold-change values (x-axis, on a logarithmic scale), *p*-value (y-axis, on a logarithmic scale). Panel (**A**) is ESI^+^, Panel (**B**) is ESI^−^, respectively Up-regulated, down-regulated, and non-differential metabolites are colored in red, blue and gray, respectively (FC > 2.0 and *p*-value < 0.05). FC, fold changed.

As shown in Figure 5, the significance of metabolite changes between these two groups were presented by univariate analysis. Volcano Plot analysis synthesized Fold Change (FC) analysis and *t*-test, which can help screen potential metabolites. The red and blue dots show metabolites that differed between these two groups under taking FC > 2.0 and *p* value < 0.05 as the selection criteria.

### 2.6. Identification of Different Metabolites

Based on the high-resolution mass measurement of molecular ions and fragmentation ions, a total of 46 different plasma metabolites from the HWL and LWL sows have been annotated and are listed in Table 5. Generally, the results showed that the main differences between HWL sows and LWL sows were the alteration of amino acids and derivatives (18), fatty acids and lipids (8), nucleotides (4), vitamin (6), bile acids (4), and others (6). Compared with LWL sows, HWL sows increased the levels of 41 plasma metabolites and decreased the levels of 5 plasma metabolites.

With the exception of creatine, all amino acids and derivative compounds were increased in the plasma of HWL when compared to LWL (Table 6). For fat metabolism, results revealed that HWL sows had a higher level of acetylcholine, lysoPC [16:0], propionic acid, valeric acid, acetylcarnitine, stearic acid, and alpha-linolenic acid compared with LWL sows, but PC(18:1(9Z)/18:1(9Z)) was decreased in HWL sows. Among the nucleosides metabolism, as compared with LWL sows, HWL sows presented an increase in the levels of allantoin, thymidine and hypoxanthine while a decrease in the level of adenosine level was observed. The levels of cholic acid, chenodeoxycholate, glycochenodeoxycholate, and glycocholic acid in the plasma of HWL sows were 3.70, 2.22, 2.08, and 1.89-fold greater, respectively, than those in LWL sows. Within vitamin metabolism, HWL sows increased the levels of N1-methyl-2-pyridone-5-carboxamide, nicotinamide, 4-pyridoxic acid, and anthranilic acid, but decreased the level of L-gulonic gamma-lactone in the plasma. In addition, the levels of 1-methylxanthine, salicyluric acid, D-quinovose, pyrocatechol, and trimethylamine N-oxide were higher in the plasma of HWL sows than LWL sows.

### 2.7. Integration of Key Metabolic Pathways

**Figure 6:**
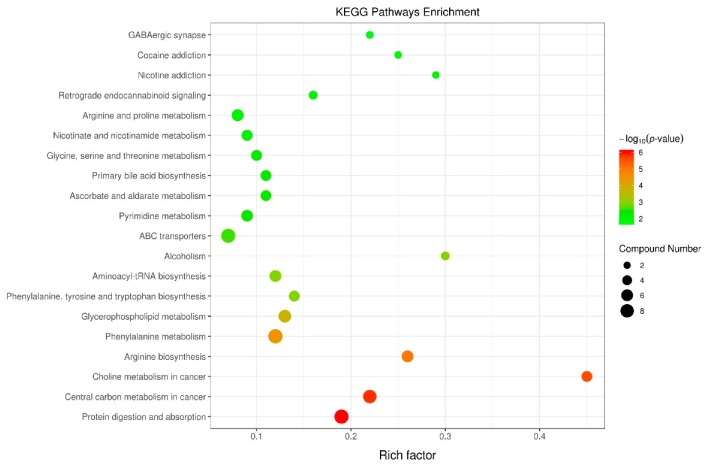
Topology analysis of metabolic pathways identified between high body weight loss (HWL, *n* = 10) and low body weight loss (LWL, *n* = 10) sows during lactation. The X-axis represents the rich factor, and the Y-axis represents the pathway. Larger sizes and darker colors represent greater pathway enrichment and higher pathway impact values, respectively.

In order to comprehensively understand the physiological change induced by excessive lactation weight loss, the Kyoto Encyclopaedia of Genes and Genomes (KEGG) pathway database was utilized for analyzing related metabolic pathways of 46 metabolites found in plasma. As shown in Figure 6, these metabolites were involved in multiple biochemical pathways, including protein digestion and absorption, central carbon metabolism, choline metabolism, amino acids metabolism and nucleotides metabolism.

## 3. Discussion

It is well known that sows exhibiting excessive weight loss during lactation represent a problem for the subsequent reproductive performance [8,17]. However, the knowledge of exact mode of action remains largely unraveled. In the present study, metabolomic based on ultrahigh-performance liquid chromatography/time-of-flight mass spectrometry was performed to clarify the underlying mechanism of excessive lactation weight loss on reproductive performance.

### 3.1. Reproductive Performance and Plasma Hormones

In the current study, HWL sows had a lower feed intake than LWL sows, which caused the increased body weight loss. Similarly, Vinsky, et al. reported that feed restriction during lactation induced excess weight loss in primiparous sows [18]. Along with higher weight loss, the WEI was prolonged in HWL sows relative to LWL sows, which is consistent with a previous study [19]. The return to estrus after weaning is dependent on the development of ovarian follicles, which is mainly regulated by metabolic mediators and circulating hormones [9,20]. High circulating IGF-1 is associated with the reduction of protein breakdown and the increase of protein synthesis [21]. As expected, present results showed that the IGF-1 concentration of HWL sows was lower than that of LWL sows, suggesting that the lower concentration of this hormone is linked to the decrease in energy intake, and thus an increased catabolism of body protein. The low concentration of IGF-1 in plasma has also been associated with impaired follicular recruitment, folliculogenesis, and reduced ovulation rate [6,22]. Interestingly, in this study, we found that plasma FGF21 concentration in HWL sows was markedly higher than in LWL sows at weaning. FGF21 is a peptide hormone, rather than a growth factor, which functions as a major metabolic regulator of glucose and lipid metabolism [23]. In line with this, a previous study reported that the concentration of FGF21 was chronically elevated from late pregnancy to lactation in high-yielding dairy cows, which was accompanied with intense lipid mobilization [24]. Moreover, FGF-21 may function as an endocrine factor to regulate body composition changes during lactation by inducing gluconeogenesis and fatty acid oxidation [25,26]. In rodents, FGF21 overexpression is shown to cause infertility, such as delayed vaginal opening, reduced mating, and anovulatory hypogonadism [27]. Furthermore, the follicular development of sows could be inhibited due to the depressed secretion of gonadotropic hormone under restricted nutritional condition or high weight loss during lactation [5,9]. Taken together, it was concluded that the disorder of hormone status induced by excessive weight loss partly contributed to failed reproductive performance in subsequent cycle.

### 3.2. Milk Composition

Previous studies show that restricted nutrient intake during lactation decreased the milk yield and composition of sows [28,29]. It was expected that milk fat content would be greater in HWL sows due to substantial fraction of fat from deposit to the milk, but the elevated milk fat content was only numerically higher. Normally, milk fat in sow ranges within 6%–8%, whereas energy restriction may increase this to very high values [30].

### 3.3. Oxidative Status

It has been well demonstrated that an increased systemic oxidative stress of sows appeared during late gestation and lactation due to the enhanced metabolic burdens [13]. In the current study, compared with LWL sows, HWL sows had higher plasma protein carbonyl and MDA concentrations but a lower T-AOC level at weaning. MDA, as a reliable marker of lipid peroxidation, is one of the toxic lipid metabolites caused by reactive oxygen species [31]. The higher MDA in HWL sows suggests that excess body weight loss led to lipid peroxidation during lactation. These findings are in accordance with the results reported in mice with calorie restriction, which demonstrated that enhanced fatty acid oxidation compared with ad libitum-feed mice [32]. Protein carbonyl is most widely utilized as the biomarker of protein oxidation [33]. The increasing oxidative damage to proteins led to chemical modification of proteins, increased protein turnover, and cell death [34], which combined with the higher protein mass loss, may imply a severe protein degradation when sows suffer from excessive lactational weight loss. Furthermore, 8-OHdG, as a major marker for oxidative damage to nucleic acids, was chosen to evaluate the DNA damage in this study. Relative to LWL sows, plasma 8-OHdG concentration in HWL sows markedly increased at weaning, indicating that HWL sows had a severe DNA damage. Collectively, these results suggest that sows with excess weight loss led to a high level of oxidative stress at weaning. Similarly, a previous study showed that cows with higher body weight loss are more sensitive to oxidative stress [14]. Taken together, oxidative stress can lead to the modification of important physiological and metabolic functions, thus the delayed WEI in sows with excessive weight loss may be ascribed to the higher oxidative stress at a certain extend.

### 3.4. Amino Acids Metabolism-Related

Amino acid metabolism could be affected in sows with excessive weight loss. Creatinine, as the indicator of muscle catabolism [35], was significantly higher in HWL sows than the LWL sows at weaning. In contrast, the creatine concentration was lower in HWL sows. It has been demonstrated that creatine is synthesized from arginine and glycine in kidney and liver, which could be phosphorylated and transported to muscle and brain and used as an energy source, and also could be broken down to creatinine [36]. Thus, the higher creatinine concentration could be resulted from a higher degradation of creatine in HWL sows. The increased creatinine concentration is associated with a greater mobilization of stored proteins and indirectly with fat level in the body mass [37], as reflected by the decreased protein and fat mass in the present study. Moreover, the level of tyrosine was significantly upregulated in HWL sows. Tyrosine, as a potent ketogenic amino acid [38], can be transformed into ketone bodies in the liver and contribute as a fuel during nutritional restriction [12]. The increased tyrosine concentration may partly be explained by tyrosine acting as a ketogenic amino acid to meet the energy requirements of lactating sows with lower feed intake. Meanwhile, glucogenic amino acids derived from muscle catabolism were important gluconeogenic substrates under malnutrition [39]. This conforms with the higher levels of amino acids and derivatives (3-phenylpropanoic acid, hippuric acid, salicylic acid, succinate, L-tryptophan, D-proline, L-phenylalanine, etc.) in HWL sows relative to LWL sows as reported in this study. The marked differences in circulating amino acid concentrations observed in the two groups may point to some possible mechanism of appetite regulation. Similarly, a hyperphagic response was found in rats fed with an imbalanced amino acid pattern diet [40]. Phenylalanine and tryptophan, which frequently linked with appetite regulation [41], were observed higher in HWL sows than in LWL sows. A pervious study has shown that improving dietary protein digestion and absorption could influence satiety through a more rapid postprandial clearance and oxidation of amino acids [42]. This is in accordance with the metabolic pathway, which showed that protein digestion and absorption were markedly changed in HWL sows. These changes could be related to the mechanism responsible for feed intake depression in HWL sows. Hippuric acid is a derivative of dietary protein catabolism [43]; the increased plasma levels of hippuric acid may be a result of increased mobilized protein from body tissues. Interestingly, sows with excessive weight loss had a higher level of plasma betaine in this study. Similarly, a previous study has shown an increase of betaine observed in growing pigs at 48 h after fasting [12]. Betaine, of dietary origin or produced from choline, is the methyl donor in methionine regeneration from homocysteine [44]. We speculate energy restriction causes an increased conversion of endogenous choline to betaine for maintaining the generally stable level of methionine. A large protein mobilization during lactation is detrimental for the sow, which needs long time to restore muscle protein in the subsequent gestation. Therefore, the optimization of protein and amino acid requirements during lactation should be concerned in future research, and understanding whether increased amino acid concentrations above the dietary recommendations may compensate for inadequate appetite.

### 3.5. Fat Metabolism-Related

Lipids, as physiological fuels, are stored in adipose tissue with low water content and high energy density [45]. Humans and animals usually break down fat to meet their energy requirements when the energy supply is restricted. The plasma concentration of NEFA, is regarded being an indicator of body fat mobilization [21]. To compensate the negative energy balance, NEFA is mobilized from adipose tissue, which was supported in the present study by elevated plasma levels of NEFA in HWL sows. In a study with dairy cattle, high NEFA levels have shown to be detrimental to embryonic survival by influencing oocyte quality, embryonic environment or both [46]. Thus, excessive fat mass loss might have a negative effect on the follicular development. Moreover, metabolites (acetylcholine and lysoPC [16:0]) related with fatty acids metabolism were increased in the plasma induced by excessive lactational weight loss. In support of this, the blood concentrations of fat related metabolites (such as fatty acids and triglycerides) can reflect the mobilization of body lipids due to negative energy balance [47]. LysoPCs are the degraded products of phosphatidylcholines, the higher level of them indicate the enhanced mobilization of phospholipids [48]. Phosphatidylcholines, as the major membrane lipids, might cause the alternation of membrane’s structure and function under the disordered phospholipid metabolism. Numerous studies have demonstrated that increased lysoPC concentrations are positively associated with endothelial dysfunction, inflammation and oxidative stress [48,49]. When the amount of acetyl-CoA exceeds the utilization of the tricarboxylic acid cycle, acetylcarnitine has a buffering function in transporting acetyl-CoA from within the mitochondria to outside [50]. In the present study, the plasma acetylcarnitine concentration in HWL sows was higher than LWL sows, indicating that a large amount of acetyl-CoA might be accumulated in the mitochondria. Thus, the results of the present study suggested that the body lipids mobilization existed in sows suffering from excessive weight loss and exhibited enhanced fatty acids oxidation.

### 3.6. Metabolic Dysfunction of Nucleotides, Bile Acids, and Vitamins

Bile acids are central to lipid and carbohydrate metabolism. In the current study, the plasma concentration of bile constituents (cholic acid, chenodeoxycholate, glycochenodeoxycholate, and glycocholic acid) were increased in HWL sows. Similarly, male mice with short-term calorie restriction showed an increased bile acids concentration in the liver, which possibly due to enhanced bile acids in enterohepatic circulation [51]. High concentrations of bile acids are toxic and increase the production of free radicals in the liver, which can cause inflammation and tissue damage [52]. In support of this, the higher plasma AST activity in HWL sows was observed in the current study. Notably, hepatic AST is distributed in the cytoplasm and mitochondria, which can be released into the blood circulation when the liver has been damaged [53]. The alteration in the level of AST might imply that HWL sows have been associated with impaired liver function due to excessive metabolic burden during lactation.

Allantoin, thymidine, and adenosine, which were markedly changed by excessive weight loss in this study, are involved in nucleoside metabolism. In human, uric acid is the end product of purine metabolism by xanthine oxidase, but it is further metabolized into allantoin in pigs [54]. A higher content of allantoin is associated with metabolic diseases, such as gout, diabetes, and kidney stones [55]. In the current study, the increased plasma allantoin level of HWL sows might suggest the metabolic disruption of purines. In accordance with our result, pigs fed with a nutrient deficient maize diet also showed a higher level of allantoin [36]. Adenosine is a ubiquitous nucleoside which serves as a building block for nucleic acids and energy storage molecules, enzyme’s substrate and neuromodulator of cellular activity [56]. The observed decrease in adenosine abundance of HWL sows suggests that more ATP was produced to maintain energy need. NAD is a coenzyme that plays a vital role in redox reaction, transferring electrons to NAD^+^ by NADH, as part of β-oxidation, glycolysis, and the TCA cycle [10], and the large decrease in nicotinamide abundance could suggest a key metabolic difference between HWL and LWL sows. These results indicate that sows with excess lactational weight loss show a higher turnover of muscle and fat tissue to allow adequate metabolism into vital organs than into the reproductive organ.

## 4. Materials and Methods

### 4.1. Animals, Diets, and Experiment Design

The experiment was approved and conducted under the supervision of the Care and Use Committee of Sichuan Agricultural University and followed the current laws of animal protection (Ethic Approval Code: SCAUAC201408-3). A total of 64 multiparous Yorkshire sows (parity 3 to 4) were used in this study (no full-sibs were included). On day 110 of pregnancy, sows were moved to the farrowing room and were kept in individual farrowing crates thereafter. Sows were fed the same commercial lactation diet until day 21 of lactation, and feed intake was increased by 1 kg/day from farrowing to day 5 of lactation, to reach ad libitum feeding. The lactation diet was in meal form and met or exceeded the nutrient requirements of lactating sows as recommended by the NRC (2012) as shown in Table 6. The feed intake of each sow during lactation was recorded daily, and average daily feed intake was calculated. The piglets had no access to creep feed, but both sows and piglets were offered water ad libitum. The ambient temperature of the sow house was maintained between 22 °C and 25 °C using draught fan and waterfall wall. All units received medical and management advice from the same veterinary consulting group. Based on the median of the percentage of weight loss during lactation, sows were retrospectively divided in a low weight loss (LWL, <10.15% of body weight, *n* = 32) or high weight loss (HWL, >10.15% of body weight, *n* = 32) groups. Previous study has demonstrated that sows with lactational weight loss > 10% had a negative effect on subsequent reproductive performance [3]. Only 20 sows (*n* = 10 for LWL, average body weight loss was 2.89%; and *n* = 10 for HWL, average body weight loss was 12.65%) were used to study effects of lactation weight loss on plasma metabolome, reproductive hormone, and antioxidant parameters.

### 4.2. Sampling and Data Collection

The body weight (BW) and backfat thickness of sows were measured in the morning (8:00) on day 110 of gestation and day 21 of lactation, while sow BW after farrowing was calculated by deduction of total litter weights of piglets and placenta from BW of sows at day 110 of gestation. Backfat thickness was measured at 65 mm from the midline of the 10th rib using an ultrasonic device (Renco Lean-Meater series 12, Renco Corporation, MN, USA). The duration of farrowing (min.), total number of piglets born, including the number of piglets born alive, number of mummified fetuses, and number of stillborn, were recorded according to Che, et al. [57]. All piglets were weighed at parturition, cross-fostered beyond 24 h and weighed weekly for calculating average daily weight gain (ADG). Estrus detection was measured two times per day (09:00 and 15:00) beginning 2 days after weaning using fence line contact with a mature teaser boar. When sows exhibited the standing reflex in the presence of the back-pressure test, they were considered to be in estrus and the time was used to calculate the WEI.

Blood samples (10 mL) were collected from sows at farrowing and before the morning meal on day 21 of lactation (at weaning). Samples were collected in heparinized tubes and plasma was harvested by centrifuging at 2550× *g* for 10 min at 4 °C, then stored at −80 °C until analysis. The Colostrum samples (approximately 30 mL) were collected from each sow within 2 h after onset of farrowing and milk samples (approximately 30 mL) were collected at day 7 of lactation after 0.3 mL oxytocin (Ocytovem, CEVA, Santé Animale, Libourne, France). Colostrum and milk samples were immediately filtered through gauze, aliquoted and stored at −20 °C immediately prior to subsequent analysis.

### 4.3. Determination of Colostrum and Milk Composition

Frozen samples (*n* = 10 for each group) were thawed at 4 °C, and 18 mL of each sample was used for milk composition analysis. The fat, protein, lactose, dry matter (DM), and urea nitrogen content were measured using a milk composition analyzer (Milkoscan 4000; Foss MilkoScan, Hillerød, Denmark) as described by Che, et al. [57].

### 4.4. Measurement of Antioxidant Parameters in the Plasma

Plasma GSH-Px, MDA, T-SOD, T-AOC, and protein carbonyl were determined using specific assay kits (Nanjing Jiancheng Institute, Jiangsu, China). Plasma concentration of MDA was quantified using thiobarbituric acid method according to Hou, et al. [58]. Plasma T-SOD and T-AOC were quantified as described by Hu, et al. [31]. Plasma GSH-Px concentration was determined using the method reported by Zhang, et al. [59]. Plasma protein carbonyl concentration was measured according to the method described by Pialoux, et al. [60]. Plasma 8-OHdG concentration was determined using ELISA kit (Cell Biolabs, San Diego, CA, USA) according to the manufacturer’s protocol. The sensitivity of this assay was 0.078 ng/mL. All plasma samples were measured in duplicate and the mean values were used for statistical analysis.

### 4.5. Measurement of Biochemical Parameters and Hormones in the Plasma

The plasma concentrations of urea, total protein (TP), alanine aminotransferase (ALT), albumin (ALB), aspartate aminotransferase (AST), lactate, triglyceride (TG), non-esterified fatty acid (NEFA), glutamyl aminotransferase (GGT), and glucose (GLU) were measured by automatic biochemical analyzer (Model 7020, Hitachi, Tokyo, Japan) according to corresponding commercial kits (Sichuan Maker Biotechnology Inc., Chengdu, China). There was less than 5% variation of intra-assay and inter-assay coefficients for each assay. Commercial enzyme-linked immunosorbent assay kits (BioVendor, Cat. No. RD291108200R) were performed to determine the plasma concentration of FGF21, and the minimal detectable concentration is 18.4 pg/mL. The intra- and inter-assay coefficients of variation were less than 8.4% and less than 8.7%, respectively. Plasma IGF-1 concentrations were determined using ELISA kits (CUSABIO Biotech Co., Ltd., Wuhan, China) according to the manufacturer’s protocol. Both intra and inter-assay coefficients of variation were less than 8.0%. Leptin was measured by ELISA kit (CUSABIO Biotech Co., Ltd., Wuhan, China). The sensitivity of this assay was 0.125 ng/mL. Intra- and inter-assay CVs were less than 15 %. All plasma samples were measured in duplicate and the mean values were used for statistical analysis.

### 4.6. Metabolomics Based on Ultra-High-Performance Liquid Chromatography Time-of-Flight/Mass Spectrometry

Plasma samples were slowly thawed at 4 °C, and from each sample a 100 μL aliquot was taken and added to 400 μL of a pre-cooled methanol/acetonitrile solution (1:1, *v*/*v*). Samples were then vortex mixed and maintained at −20 °C for 60 min followed by centrifugation at 14,000× *g* and 4 °C for 20 min. The supernatant fraction was collected and dried. The dried metabolites were dissolved by adding 100 μL of aqueous acetonitrile (acetonitrile: water = 1:1, *v*/*v*), vortex mixed, and centrifuged at 14,000× *g* and 4 °C for 15 min. The resulting supernatant was collected and analyzed. The 2 sets of treated samples were mixed in equal amounts for the preparation of quality control (QC) samples, and 5 replicates were set up to evaluate system stability over the entire experiment before testing. After the completion of sample pre-treatment, the samples were transferred to sampler vials for liquid chromatography-tandem mass spectrometry (LC/MS) analysis.

Samples after pretreatment were separated using an ultra-high-performance liquid chromatography (UHPLC) system (1290 Infinity II, Agilent Technologies, Santa Clara, CA, USA) incorporating an hydrophilic interaction liquid chromatography (HILIC) column (2.1 mm × 100 mm, 1.7 μm; Waters, Milford, MA, USA). The column temperature was 25 °C, and we used a flow rate of 0.3 mL/min. The mobile phase consisted of A (water + 25 mmol/L of ammonium acetate + 25 mmol/L of ammonia) and B (acetonitrile). The gradient elution procedure was as follows: 0–1 min, 95%B; 1–14 min, 95% to 65%B; 14–16 min, 65% to 40%B; 16–18 min, 40%B; 18–18.1 min, 40% to 95%B; and 18.1–23 min, 95%B. The autosampler was maintained at 4 °C and the injection volume was 2 μL. The QC samples were inserted into the samples to monitor system stability and data quality.

The samples were analyzed using a triple time-of-flight (TOF) 5600 + system (AB/SCIEX, Framingham, MA, USA) equipped with an electrospray ionization source used in positive and negative ion modes. The mass spectrometry detection variables were as follows: Gas 1, 0.4137 MPa; gas 2, 0.4137 MPa; curtain gas, 0.20685 MPa; ion source temperature, 600 °C; ionization voltage, ± 5500 V; TOF-MS scan range, 60–1000 m/z; precursor ion scan range, 25–1000 m/z; scan accumulation time, 0.2 s/spectrum; precursor ion scan accumulation time, 0.05 s/spectrum; declustering potential, 60 V; and collision energy, 35 ± 15 eV. Tandem mass spectrometry data were acquired in the information-dependent acquisition mode, and high sensitivity modes were used.

### 4.7. Data Processing and Statistical Analyses

The raw data were converted into the mzXML format using ProteoWizard [61], and then peak alignment, retention time correction, and peak area extraction were performed using the R package XCMS [62]. For the data extracted using XCMS, ion peak data for which >50% of the data were missing within a group were deleted. After the data had been pre-processed by pareto-scaling, pattern recognition was performed using SIMCA-P software (version 14.1, Umetrics, Umea, Sweden), consisting of unsupervised PCA and supervised OPLS-DA. Principal component analysis was used to determine intra-group aggregation and inter-group separation tendencies, whereas OPLS-DA was performed to further determine inter-group differences. The OPLS-DA models were validated based on interpretation of variation in Y (R^2^Y) and forecast ability based on the model (Q^2^) in cross-validation and permutation tests by applying 200 iterations. When 1 ≥ R^2^Y and Q^2^ ≥ 0.4, the models were determined to be stable and reliable [63]. In addition, a Q^2^ intercept < 0.05 from the permutation test was used to verify that there was no overfitting, and univariate analysis was performed, including Student’s *t*-test and fold change analysis.

Significantly differential metabolites were screened using variable importance in projection (VIP) scores (VIP > 1) obtained from the OPLS-DA model and *P* values (*p* < 0.05). Identification of differential metabolites was carried out by searching an in-house standard MS/MS library and the online database METLIN [64] using MS/MS spectra or exact mass data [62]. The in-house library contains MS/MS spectra of approximately 800 compounds, which were obtained from standards. The MS/MS spectra matching score was calculated using the dot-product algorithm and the score cutoff was set as 0.8. The MS/MS spectra that could not be matched to any of those in the in-house library were searched in online databases. Mass error was set within 25 ppm. Moreover, pathway analysis data were processed and analyzed using MetaboAnalyst 4.0 [65].

The protein and fat mass of sows were calculated according to Dourmad, et al. [66], as follows: lipids (kg) = –26.4 + 0.221 × EBW + 1.331 × P_2_; protein (kg) = 2.28 + 0.178 × EBW − 0.333 × P_2_, where EBW (kg) represents the sow empty live weight (EBW = 0.905 × BW^1.1013^, BW = live weight in kg) and P_2_ (mm) = backfat thickness at the last rib. The individual sow was used as the experimental unit for all response variables. Data were analyzed by an independent-samples *t*-test using SPSS 21.0 (IBM SPSS Company, Chicago, IL, USA). Results were considered significant at *p* < 0.05 and as trend at 0.05 < *p* < 0.1.

## 5. Conclusions

In summary, this study demonstrates that sows with excessive lactational weight loss is associated with a noticeable disorder in amino acids metabolism and fatty acid oxidation metabolism due to protein breakdown and lipid mobilization, which leads to a serious oxidative damage and metabolic dysfunction at the end of lactation, thus delaying the weaning to estrus interval. We recommend directing further research towards optimizing the diet composition in early lactation and post-weaning diet based on these different metabolites in order to improve the appetite of lactating sows and the follicular development of sows with excessive weight loss.

## Figures and Tables

**Table 1:** Reproductive performance of sows with a low or high lactational weight loss.

Item	LWL	HWL	*p*-Value
Total born piglets, no.	15.54 ± 0.98	15.80 ± 0.96	0.854
Live born piglets, no.	14.46 ± 0.79	15.00 ± 0.91	0.958
Stillborn piglets, no.	0.92 ± 0.31	0.60 ± 0.27	0.539
Mummified fetuses, no.	0.15 ± 0.10	0.20 ± 0.14	0.749
Total born			
Litter birth weight, kg	20.97 ± 1.26	19.22 ± 1.40	0.378
Mean piglet BW, kg	1.37 ± 0.06	1.24 ± 0.07	0.332
Live born			
Litter birth weight, kg	19.74 ± 1.00	18.59 ± 1.35	0.492
Mean piglet BW, kg	1.39 ± 0.06	1.25 ± 0.07	0.306
Duration of farrowing, min	234.38 ± 20.30	250.47 ± 24.47	0.617
Placenta weight, kg	4.00 ± 0.19	4.17 ± 0.35	0.695
Litter size after cross-foster, no.	11.93 ± 0.55	12.30 ± 0.23	0.535
ADFI (Day 0–21), kg/day	5.22 ± 0.09	4.96 ± 0.08	0.037
Litter weight at weaning, kg	60.70 ± 3.87	67.38 ± 7.47	0.478
Piglet ADG (Day 0–21), g/day	246 ± 10	233 ± 12	0.433

Data are expressed as the mean ± SEM. Sows were regarded as the experimental units, *n* = 10 for each group. LWL, low weight loss; HWL, high weight loss; ADFI, average daily feed intake; ADG, average daily growth.

**Table 2:** Sow live weight, backfat, and calculated protein and fat mass during lactation of sows with a low or high lactational weight loss.

Item	LWL	HWL	*p*-Value
Body weight at gestation 110 day, kg	282.67 ± 7.62	283.76 ± 4.50	0.899
At parturition			
Body weight, kg	259.13 ± 7.36	263.82 ± 4.16	0.572
Back fat depth, mm	18.40 ± 0.90	18.94 ± 0.66	0.626
Fat mass, kg	53.40 ± 2.37	54.53 ± 1.20	0.675
Protein mass, kg	40.40 ± 1.14	41.18 ± 0.76	0.567
At weaning			
Body weight, kg	251.67 ± 6.88	230.41 ± 3.64	0.008
Back fat depth, mm	15.07 ± 0.87	13.41 ± 0.67	0.140
Fat mass, kg	47.07 ± 2.15	40.35 ± 1.45	0.013
Protein mass, kg	40.27 ± 1.11	37.06 ± 0.56	0.018
Lactation BW loss, kg	7.67 ± 1.94	33.41 ± 1.60	<0.001
Lactation back fat loss, mm	3.50 ± 0.25	5.35 ± 0.77	0.033
Lactation BW loss, %	2.89 ± 0.74	12.65 ± 0.53	<0.001
Calculated sow fat mass			
Lactation fat loss, kg	6.29 ± 0.45	14.22 ± 1.05	<0.001
Lactation fat loss, %	11.86 ± 0.75	26.10 ± 1.80	<0.001
Calculated sow protein mass			
Lactation protein loss, kg	0.14 ± 0.37	3.93 ± 0.39	<0.001
Lactation protein loss, %	0.27 ± 0.90	9.44 ± 0.82	<0.001
WEI, h	112.00 ± 6.49	132.71 ± 7.16	0.029

Data are expressed as the mean ± SEM. Sows were regarded as the experimental units, *n* = 10 for each group. LWL, low weight loss; HWL, high weight loss; WEI, weaning to estrus interval.

**Table 3:** Colostrum and milk composition of sows with a low or high lactational weight loss.

Item	LWL	HWL	*p*-Value
Colostrum			
Fat, %	4.42 ± 0.38	4.65 ± 0.39	0.674
Protein, %	19.24 ± 0.95	19.85 ± 0.98	0.656
Lactose, %	2.71 ± 0.20	2.60 ± 0.21	0.702
Total solids, %	27.07 ± 0.97	27.79 ± 1.19	0.637
Urea nitrogen, mg/dl	28.00 ± 1.14	26.72 ± 1.94	0.573
Milk			
Fat, %	8.14 ± 0.34	8.86 ± 0.74	0.379
Protein, %	5.68 ± 0.22	5.85 ± 0.14	0.502
Lactose, %	5.35 ± 0.30	5.69 ± 0.20	0.342
Total solids, %	19.86 ± 0.73	21.10 ± 0.74	0.237
Urea nitrogen, mg/dl	24.77 ± 1.00	26.10 ± 1.34	0.429

Data are expressed as the mean ± SEM. Sows were regarded as the experimental units, *n* = 10 for each group. LWL, low weight loss; HWL, high weight loss.

**Table 4:** Plasma biochemical profiles at farrowing and weaning in sows with a low or high lactational weight loss.

Item	LWL	HWL	*p*-Value
Farrowing			
GLU, mmol/L	3.65 ± 0.51	2.80 ± 0.43	0.207
LAC, mmol/L	2.24 ± 0.20	2.47 ± 0.15	0.368
NEFA, μmol/L	809.36 ± 71.18	848.00 ± 117.00	0.750
TG, μmol/L	251.15 ± 22.88	241.17 ± 32.25	0.732
UREA, mmol/L	3.48 ± 0.18	3.65 ± 0.21	0.537
TP, g/L	74.20 ± 2.44	73.26 ± 1.86	0.764
ALB, g/L	38.84 ± 1.00	38.58 ± 1.50	0.884
Weaning			
GLU, mmol/L	4.15 ± 0.57	4.86 ± 0.63	0.200
LAC, mmol/L	2.45 ± 0.24	2.02 ± 0.19	0.210
NEFA, μmol/L	55.00 ± 8.11	80.55 ± 9.52	0.025
TG, μmol/L	342.64 ± 75.13	428.00 ± 76.32	0.450
UREA, mmol/L	7.02 ± 0.46	6.04 ± 0.77	0.148
TP, g/L	83.25 ± 1.70	80.66 ± 1.68	0.339
ALB, g/L	38.79 ± 0.90	38.51 ± 1.85	0.902

Data are expressed as the mean ± SEM. Sows were regarded as the experimental units, *n* = 10 for each group. GLU, glucose; LAC, lactose; NEFA, non-esterified fatty acid; TG, triglyceride; TP, total protein; ALB, albumin; LWL, low weight loss; HWL, high weight loss.

**Table 5:** List of plasma metabolites discriminating among sows with low and high lactational weight loss.

Name	Ion	FC	*p*-Value	M-to-Z Ratio	RT (min)
Amino acid metabolism-related					
3,4-Dihydroxybenzoate	[M − H]^−^	2.22	0.001	153.0189	0.69
3-Phenylpropanoic acid	[M − H]^−^	1.67	0.018	149.0606	2.24
Betaine	[M + H]^+^	1.32	0.002	118.0856	8.58
Creatine	[M + H]^+^	0.58	0.023	132.0760	11.06
Creatinine	[M + H]^+^	1.23	0.017	114.0653	5.22
Hippuric acid	[M + H]^+^	1.59	0.027	180.0647	6.28
L-Carnosine	[M + H]^+^	1.69	0.001	227.1134	13.40
L-Citrulline	[M + H]^+^	1.47	0.000	176.1024	12.46
L-Phenylalanine	[M + H]^+^	1.28	0.048	166.0859	7.94
L-Tyrosine	[M − H]^−^	1.33	0.019	180.0667	9.38
Salicylic acid	[M − H]^−^	1.85	0.001	137.0240	1.01
Succinate	[M − H]^−^	1.39	0.003	117.0190	12.53
Tyramine	[M + H − H_2_O]^+^	1.28	0.046	120.0800	7.93
Hydroxyisocaproic acid	[M − H]^−^	1.47	0.006	131.0710	3.45
2-Oxoadipic acid	[M − H_2_O − H]^−^	1.04	0.088	141.0170	10.81
L-Tryptophan	[M − H]^−^	1.27	0.064	203.0827	7.91
D-Proline	[M − H]^−^	1.27	0.052	114.0559	9.93
3-Indolepropionic acid	[M + H]^+^	2.44	0.007	190.0854	3.22
Fat metabolism-related					
Acetylcholine	[M + H]^+^	1.56	0.001	146.1169	11.96
LysoPC [16:0]	[M + H]^+^	1.37	0.013	496.3382	5.80
Propionic acid	[M + CH_3_COO]^−^	1.96	0.030	133.0499	3.38
Valeric acid	[M − H]^−^	1.45	0.025	101.0604	3.29
PC(18:1(9Z)/18:1(9Z))	[M − H + 2Na]^+^	0.63	0.042	830.5659	1.41
Acetylcarnitine	[M + H]^+^	1.25	0.079	204.1226	9.66
Stearic acid	[M − H]^−^	2.13	0.079	283.2644	1.68
alpha-Linolenic acid	[M − H]^−^	1.20	0.063	277.2172	1.25
Nucleoside metabolism-related					
Adenosine	[M + H]^+^	0.49	0.000	268.1031	5.27
Allantoin	[M + H]^+^	1.49	0.000	159.0505	5.63
Thymidine	[M − H]^−^	2.00	0.023	241.0832	3.03
Hypoxanthine	[M − H]^−^	1.22	0.069	135.0310	5.20
Vitamin metabolism-related					
N1-Methyl-2-pyridone-5-carboxamide	[M − H]^−^	1.43	0.016	151.0509	2.47
Nicotinamide	[M + H]^+^	1.89	0.014	123.0543	1.54
4-Pyridoxic acid	[M + H]^+^	1.61	0.002	184.0597	1.11
L-Gulonic gamma-lactone	[M − H]^−^	0.69	0.041	177.0405	4.33
Anthranilic acid (Vitamin L1)	[M + H]^+^	1.37	0.075	138.0542	9.18
Bile acid metabolism-related					
Cholic acid	[M + H − H_2_O]^+^	3.70	0.003	391.2834	3.46
Chenodeoxycholate	[M − H]^−^	2.22	0.027	391.2854	4.85
Glycochenodeoxycholate	[M + H]^+^	2.08	0.013	450.3206	6.88
Glycocholic acid	[M − H]^−^	1.89	0.028	464.3015	7.75
Others					
1-Methylxanthine	[M + K − 2H]^−^	1.61	0.036	203.0018	0.66
D-Mannose	[M + NH_4_]^+^	0.79	0.042	198.0964	9.49
Salicyluric acid	[M − H]^−^	2.70	0.014	194.0457	6.98
D-Quinovose	[M + CH_3_COO]^−^	1.67	0.022	223.0823	6.61
Pyrocatechol	[M − H]^−^	1.79	0.032	109.0289	0.65
Trimethylamine N-oxide	[M + H]^+^	2.44	0.004	76.0751	10.41

FC, fold change, was calculated by dividing the mean intensity of high body weight loss sows’ plasma metabolites by the mean intensity of low body weight loss sows’ plasma metabolites; RT, retention time; PC, phosphatidylcholine; LysoPC, lysophosphatidylcholine; M-to-Z ratio, mass-to-charge ratio.

**Table 6:** Composition of lactation diet and nutrient level (air-dry basis, %).

Constituent	%	Nutritional Value	%
Corn	59.86	Metabolizable energy, Mcal/kg	3.41
Soybean meal	17.68	Crude protein	17.20
Fish meal	2.00	Lysine	1.00
Extruded soybean	6.00	Methionine	0.27
Wheat	3.59	Threonine	0.63
Soybean hull	5.00	Tryptophan	0.18
Limestone	0.87	Neutral detergent fiber	11.00
Calcium hydrophosphate	0.93	Calcium	0.90
Vitamin and mineral mix ^1^	0.63	Available phosphorus	0.45
Salt	0.40	Crude fiber	4.10
Soybean oil	2.00	Ether extract	6.10
l-Lysine HCl (98.5%)	0.25	-	-
l-Threonine (98.5%)	0.09	-	-
dl-Methionine (99%)	0.05	-	-
Choline chloride (50%)	0.15	-	-
Potassium chloride	0.50	-	-

^1^ Premix per kilogram diet: Zn 67 mg; Cu 13 mg; Fe 73 mg; Mn 33 mg; Co 0.13 mg; I 0.33 mg; Se 0.27 mg; VA 12500 UI; VD3 2000 UI; VE 60 mg; VK3 2.5 mg; VB1 2.5 mg; VB2 6.3 mg; VB3 20 mg; VB6 2.5 mg; VB12 0.03 mg; nicotinic acid 35 mg; folic acid 3.0 mg; biotin 0.3 mg.

## Supplementary Materials

The following are available online at https://www.mdpi.com/2218-1989/9/12/295/s1, Figure S1: Representative reversed-phase high performance liquid chromatography coupled to electrospray ionization quadrupole time-of-flight mass spectrometry base peak chromatograms (BPC) of plasma sample analyzed in positive (A) and negative (B) electrospray ionization (ESI) modes. Details on the separation conditions are given in the experimental section, Figure S2: PCA scores plot comparing HWL with LWL sows in ESI^+^ and ESI^−^ metabolomics profiles of plasma (Panel A and B are ESI^+^ and ESI^−^, respectively). LWL, low body weight loss; HWL, high body weight loss. Table S1: The values of R^2^X, R^2^Y, and Q^2^ in OPLS-DA model.

## References

[1] Eissen J.J., Apeldoorn E.J., Kanis E., Verstegen M.W., de Greef K.H. (2003). The importance of a high feed intake during lactation of primiparous sows nursing large litters. J. Anim. Sci..

[2] Koketsu Y., Dial G.D., Pettigrew J.E., King V.L. (1996). Feed intake pattern during lactation and subsequent reproductive performance of sows. J. Anim. Sci..

[3] Thaker M.Y., Bilkei G. (2005). Lactation weight loss influences subsequent reproductive performance of sows. Anim. Reprod. Sci..

[4] Strathe A.V., Bruun T.S., Hansen C.F. (2017). Sows with high milk production had both a high feed intake and high body mobilization. Animal.

[5] Schenkel A.C., Bernardi M.L., Bortolozzo F.P., Wentz I. (2010). Body reserve mobilization during lactation in first parity sows and its effect on second litter size. Livest. Sci..

[6] Prunier A., Quesnel H. (2000). Influence of the nutritional status on ovarian development in female pigs. Anim. Reprod. Sci..

[7] Hoving L.L., Soede N.M., Feitsma H., Kemp B. (2012). Lactation Weight Loss in Primiparous Sows: Consequences for Embryo Survival and Progesterone and Relations with Metabolic Profiles. Reprod. Domest. Anim..

[8] Wientjes J.G., Soede N.M., Knol E.F., van den Brand H., Kemp B. (2013). Piglet birth weight and litter uniformity: Effects of weaning-to-pregnancy interval and body condition changes in sows of different parities and crossbred lines. J. Anim. Sci..

[9] Quesnel H., Rodriguez-Martinez H., Vallet J., Ziecik A. (2009). Nutritional and lactational effects on follicular development in the pig. Control Pig Reprod. VIII.

[10] Ramsay T.G., Stoll M.J., Shannon A.E., Blomberg L.A. (2018). Metabolomic analysis of longissimus from underperforming piglets relative to piglets with normal preweaning growth. J. Anim. Sci. Biotechnol..

[11] Guijas C., Montenegro-Burke J.R., Warth B., Spilker M.E., Siuzdak G. (2018). Metabolomics activity screening for identifying metabolites that modulate phenotype. Nat. Biotechnol..

[12] Liu H., Chen Y., Ming D., Wang J., Li Z., Ma X., Wang J., van Milgen J., Wang F. (2018). Integrative analysis of indirect calorimetry and metabolomics profiling reveals alterations in energy metabolism between fed and fasted pigs. J. Anim. Sci. Biotechnol..

[13] Berchieri-Ronchi C.B., Kim S.W., Zhao Y., Correa C.R., Yeum K.J., Ferreira A.L. (2011). Oxidative stress status of highly prolific sows during gestation and lactation. Animal.

[14] Bernabucci U., Ronchi B., Lacetera N., Nardone A. (2005). Influence of body condition score on relationships between metabolic status and oxidative stress in periparturient dairy cows. J. Dairy Sci..

[15] Keaney J.F., Larson M.G., Vasan R.S., Wilson P.W.F., Lipinska I., Corey D., Massaro J.M., Sutherland P., Vita J.A., Benjamin E.J. (2003). Obesity and systemic oxidative stress—Clinical correlates of oxidative stress in the Framingham Study. Arterioscl. Throm. Vas..

[16] Morrow J.D. (2003). Is oxidant stress a connection between obesity and atherosclerosis?. Arterioscl. Throm. Vas..

[17] Rojkittikhun T., Einarsson S., Uvnas-Moberg K., Edqvist L.E. (1993). Body weight loss during lactation in relation to energy and protein metabolism in standard-fed primiparous sows. Zentralbl. Veterinarmed. A.

[18] Vinsky M.D., Novak S., Dixon W.T., Dyck M.K., Foxcroft G.R. (2006). Nutritional restriction in lactating primiparous sows selectively affects female embryo survival and overall litter development. Reprod. Fertil. Dev..

[19] Kemp B., Soede N.M. (2012). Should weaning be the start of the reproductive cycle in hyper-prolific sows? A physiological view. Reprod. Domest. Anim..

[20] Hulten F., Valros A., Rundgren M., Einarsson S. (2002). Reproductive endocrinology and postweaning performance in the multiparous sow. Part 1. Influence of metabolic status during lactation. Theriogenology.

[21] Mosnier E., Etienne M., Ramaekers P., Père M.C. (2010). The metabolic status during the peri partum period affects the voluntary feed intake and the metabolism of the lactating multiparous sow. Livest. Sci..

[22] Costermans N.G.J., Teerds K.J., Middelkoop A., Roelen B.A.J., Schoevers E.J., van Tol H.T.A., Laurenssen B., Koopmanschap R.E., Zhao Y., Blokland M. (2019). Consequences of negative energy balance on follicular development and oocyte quality in primiparous sows. Biol. Reprod..

[23] Fisher F.M., Maratos-Flier E. (2016). Understanding the Physiology of FGF21. Annu. Rev. Physiol..

[24] Schoenberg K.M., Giesy S.L., Harvatine K.J., Waldron M.R., Cheng C., Kharitonenkov A., Boisclair Y.R. (2011). Plasma FGF21 is elevated by the intense lipid mobilization of lactation. Endocrinology.

[25] Bornstein S., Brown S.A., Le P.T., Wang X., DeMambro V., Horowitz M.C., MacDougald O., Baron R., Lotinun S., Karsenty G. (2014). FGF21 and skeletal remodeling during and after lactation in C57BL/6J mice. Endocrinology.

[26] Zhuo Y., Hua L., Feng B., Jiang X.M., Li J., Jiang D.D., Huang X.H., Zhu Y.G., Li Z., Yan L.J. (2019). Fibroblast growth factor 21 coordinates adiponectin to mediate the beneficial effects of low-protein diet on primordial follicle reserve. Ebiomedicine.

[27] Owen B.M., Bookout A.L., Ding X., Lin V.Y., Atkin S.D., Gautron L., Kliewer S.A., Mangelsdorf D.J. (2013). FGF21 contributes to neuroendocrine control of female reproduction. Nat. Med..

[28] Beyer M., Jentsch W., Kuhla S., Wittenburg H., Kreienbring F., Scholze H., Rudolph P.E., Metges C.C. (2007). Effects of dietary energy intake during gestation and lactation on milk yield and composition of first, second and fourth parity sows. Arch. Anim. Nutr..

[29] Noblet J., Etienne M. (1986). Effect of energy level in lactating sows on yield and composition of milk and nutrient balance of piglets. J. Anim. Sci..

[30] Revell D.K., Williams I.H., Mullan B.P., Ranford J.L., Smits R.J. (1998). Body composition at farrowing and nutrition during lactation affect the performance of primiparous sows: II. Milk composition, milk yield, and pig growth. J. Anim. Sci..

[31] Hu L., Peng X., Qin L., Wang R., Fang Z., Lin Y., Xu S., Feng B., Wu D., Che L. (2018). Dietary nucleotides supplementation during the suckling period improves the antioxidative ability of neonates with intrauterine growth retardation when using a pig model. RSC Adv..

[32] Cao S.X., Dhahbi J.M., Mote P.L., Spindler S.R. (2001). Genomic profiling of short- and long-term caloric restriction effects in the liver of aging mice. Proc. Natl. Acad. Sci. USA.

[33] Dalle-Donne I., Rossi R., Giustarini D., Milzani A., Colombo R. (2003). Protein carbonyl groups as biomarkers of oxidative stress. Clin. Chim. Acta.

[34] Simm A., Brömme H.J. (2005). Reactive oxygen species (ROS) and aging: Do we need them—can we measure them—Should we block them?. Signal Transduct..

[35] Yang Y.X., Heo S., Jin Z., Yun J.H., Choi J.Y., Yoon S.Y., Park M.S., Yang B.K., Chae B.J. (2009). Effects of lysine intake during late gestation and lactation on blood metabolites, hormones, milk composition and reproductive performance in primiparous and multiparous sows. Anim. Reprod. Sci..

[36] Jiang P., Stanstrup J., Thymann T., Sangild P.T., Dragsted L.O. (2016). Progressive Changes in the Plasma Metabolome during Malnutrition in Juvenile Pigs. J. Proteome Res..

[37] Vanniekerk B.D.H., Reid J.T., Paladines O.L., Bensadoun A. (1963). Urinary Creatinine as an Index of Body Composition. J. Nutr..

[38] Balasse E.O., Fery F. (1989). Ketone-Body Production and Disposal—Effects of Fasting, Diabetes, and Exercise. Diabetes Metab. Rev..

[39] Ruderman N.B. (1975). Muscle Amino-Acid Metabolism and Gluconeogenesis. Annu. Rev. Med..

[40] Sanahuja J.C., Rio M.E., Lede M.N. (1965). Decrease in Appetite and Biochemical Changes in Amino Acid Imbalance in the Rat. J. Nutr..

[41] Hill A., Blundell J. (1988). Role of amino acids in appetite control in man. Amino Acid Availability and Brain Function in Health and Disease.

[42] Hall W.L., Millward D.J., Long S.J., Morgan L.M. (2003). Casein and whey exert different effects on plasma amino acid profiles, gastrointestinal hormone secretion and appetite. Br. J. Nutr..

[43] Rempel L.A., Miles J.R., Oliver W.T., Broeckling C.D. (2016). Non-targeted Plasma Metabolome of Early and Late Lactation Gilts. Front. Mol. Biosci..

[44] Hollenbeck C.B. (2012). An introduction to the nutrition and metabolism of choline. Cent. Nerv. Syst. Agents Med. Chem..

[45] Guo C., Xue Y., Seddik H.-E., Yin Y., Hu F., Mao S. (2019). Dynamic Changes of Plasma Metabolome in Response to Severe Feed Restriction in Pregnant Ewes. Metabolites.

[46] Leroy J.L., Van Soom A., Opsomer G., Bols P.E. (2008). The consequences of metabolic changes in high-yielding dairy cows on oocyte and embryo quality. Animal.

[47] McCue M.D. (2010). Starvation physiology: Reviewing the different strategies animals use to survive a common challenge. Comp. Biochem. Physiol. Part A Mol. Integr. Physiol..

[48] Wellner N., Diep T.A., Janfelt C., Hansen H.S. (2013). N-acylation of phosphatidylethanolamine and its biological functions in mammals. BBA Mol. Cell Biol. Lipids.

[49] Greig F.H., Kennedy S., Spickett C.M. (2012). Physiological effects of oxidized phospholipids and their cellular signaling mechanisms in inflammation. Free Radic Biol Med..

[50] Lopaschuk G.D., Gamble J. (1994). Acetyl-Coa Carboxylase—An Important Regulator of Fatty-Acid Oxidation in the Heart. Can. J. Physiol. Pharmacol..

[51] Fu Z.D., Klaassen C.D. (2013). Increased bile acids in enterohepatic circulation by short-term calorie restriction in male mice. Toxicol. Appl. Pharmacol..

[52] Bobe G., Young J.W., Beitz D.C. (2004). Invited review: Pathology, etiology, prevention, and treatment of fatty liver in dairy cows. J. Dairy Sci..

[53] Feng C., Bai K., Wang A., Ge X., Zhao Y., Zhang L., Wang T. (2018). Effects of dimethylglycine sodium salt supplementation on growth performance, hepatic antioxidant capacity, and mitochondria-related gene expression in weanling piglets born with low birth weight. J. Anim. Sci..

[54] Huang C.C., Lou B.S., Hsu F.L., Hou C.C. (2014). Use of urinary metabolomics to evaluate the effect of hyperuricemia on the kidney. Food Chem. Toxicol..

[55] Xue Y., Guo C., Hu F., Liu J., Mao S. (2018). Hepatic Metabolic Profile Reveals the Adaptive Mechanisms of Ewes to Severe Undernutrition during Late Gestation. Metabolites.

[56] Porkka-Heiskanen T., Kalinchuk A.V. (2011). Adenosine, energy metabolism and sleep homeostasis. Sleep Med. Rev..

[57] Che L., Hu L., Wu C., Xu Q., Zhou Q., Peng X., Fang Z., Lin Y., Xu S., Feng B. (2019). Effects of increased energy and amino acid intake in late gestation on reproductive performance, milk composition, metabolic, and redox status of sows. J. Anim. Sci..

[58] Hou X., Wang T., Ahmad H., Xu Z. (2017). Ameliorative effect of ampelopsin on LPS-induced acute phase response in piglets. J. Funct. Foods.

[59] Zhang X.-D., Zhu Y.-F., Cai L.-S., Wu T.-X. (2008). Effects of fasting on the meat quality and antioxidant defenses of market-size farmed large yellow croaker (*Pseudosciaena crocea*). Aquaculture.

[60] Pialoux V., Mounier R., Rock E., Mazur A., Schmitt L., Richalet J.P., Robach P., Coudert J., Fellmann N. (2009). Effects of acute hypoxic exposure on prooxidant/antioxidant balance in elite endurance athletes. Int. J. Sports Med..

[61] Chambers M.C., Maclean B., Burke R., Amodei D., Ruderman D.L., Neumann S., Gatto L., Fischer B., Pratt B., Egertson J. (2012). A cross-platform toolkit for mass spectrometry and proteomics. Nat. Biotechnol..

[62] Jia H.X., Shen X.T., Guan Y., Xu M.M., Tu J., Mo M., Xie L., Yuan J., Zhang Z., Cai S.J. (2018). Predicting the pathological response to neoadjuvant chemoradiation using untargeted metabolomics in locally advanced rectal cancer. Radiother. Oncol..

[63] Westerhuis J.A., Hoefsloot H.C.J., Smit S., Vis D.J., Smilde A.K., van Velzen E.J.J., van Duijnhoven J.P.M., van Dorsten F.A. (2008). Assessment of PLSDA cross validation. Metabolomics.

[64] METLIN. http://metlin.scripps.edu.

[65] MetaboAnalyst-Statistical, Functional and Integrative Analysis of Metabolomics Data. http://www.metaboanalyst.ca.

[66] Dourmad J., Etienne M., Noblet J., Causeur D. (1997). Prediction de la composition chimique des truies reproductrices a partir du poids vif et de l’epaisseur de lard dorsal. J. Rech. Porc. Fr..

