# Supplementary material for: Metabolomic Profiling Reveals the Difference on Reproductive Performance between High and Low Lactational Weight Loss Sows
Source: Metabolites. 2019 Dec 4;9(12):295. doi: 10.3390/metabo9120295 (PMC6950487; doi:10.3390/metabo9120295)
Supplement: Supplementary file 1 [file metabolites-09-00295-s001.docx]

A.

B.

**Figure S1.** Representative reversed-phase high performance liquid chromatography coupled to electrospray ionization quadrupole time-of-flight mass spectrometry base peak chromatograms (BPC) of plasma sample analyzed in positive (**A**) and negative (**B**) electrospray ionization (ESI) modes. Details on the separation conditions are given in the experimental section.

A.
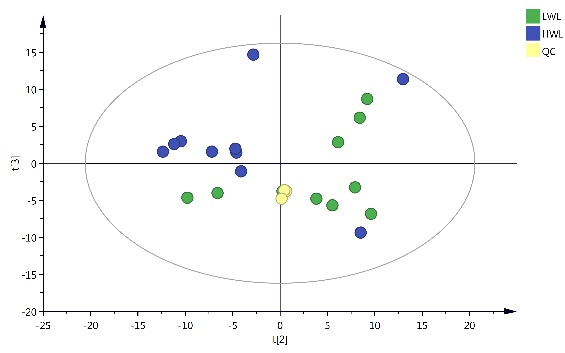
B.
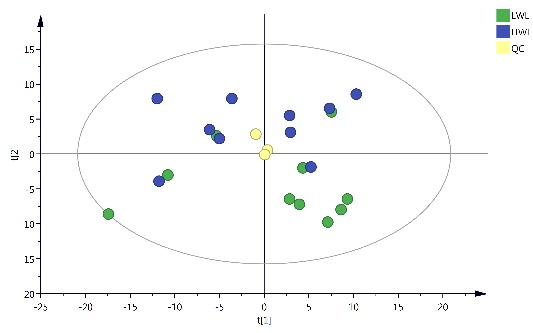


**Figure S2.** PCA scores plot comparing HWL with LWL sows in ESI^+^ (Panel **A**) and ESI^－^ (Panel **B**) metabolomics profiles of plasma. LWL, low body weight loss; HWL, high body weight loss. t[1] = first principal component. t[2] = second principal component.

**Table S1.** The values of R^2^X, R^2^Y, and Q^2^ in OPLS-DA model.

| **Model** | **R^2^X (cum)** | **R^2^Y(cum)** | **Q^2^(cum)** |
| --- | --- | --- | --- |
| ESI^+^ | 0.465 | 0.997 | 0.477 |
| ESI^－^ | 0.250 | 0.915 | 0.516 |
